# Korean Variant Archive (KOVA): a reference database of genetic variations in the Korean population

**DOI:** 10.1038/s41598-017-04642-4

**Published:** 2017-06-27

**Authors:** Sangmoon Lee, Jihae Seo, Jinman Park, Jae-Yong Nam, Ahyoung Choi, Jason S. Ignatius, Robert D. Bjornson, Jong-Hee Chae, In-Jin Jang, Sanghyuk Lee, Woong-Yang Park, Daehyun Baek, Murim Choi

**Affiliations:** 1 Department of Biomedical Sciences, Seoul National University College of Medicine, Seoul, 03080 Republic of Korea; 2 Ewha Research Center for Systems Biology (ERCSB), Ewha Womans University, Seoul, 03760 Republic of Korea; 3 Center for RNA Research, Institute for Basic Science, Seoul, 08826 Republic of Korea; 4 School of Biological Sciences, Seoul National University, Seoul, 08826 Republic of Korea; 5 Samsung Genome Institute, Samsung Medical Center, Seoul, 06351 Republic of Korea; 6 Department of Health Sciences and Technology, Samsung Advanced Institute of Science and Heath Technology, Sungkyunkwan University, Seoul, 06351 Republic of Korea; 7 Department of Bio-Information Science, Ewha Womans University, Seoul, 03760 Republic of Korea; 8 Yale Center for Research Computing, Yale University, New Haven, CT 06511 USA; 9 Department of Computer Science and Yale Center for Research Computing, Yale University, New Haven, CT 06511 USA; 10 Department of Pediatrics, Seoul National University Children’s Hospital, Seoul National University College of Medicine, Seoul, 03080 Republic of Korea; 11 Department of Clinical Pharmacology and Therapeutics, Seoul National University College of Medicine and Seoul National University Hospital, Seoul, 03080 Republic of Korea; 12 Department of Molecular Cell Biology, Sungkyunkwan University School of Medicine, Suwon, 16419 Republic of Korea; 13 Bioinformatics Institute, Seoul National University, Seoul, 08826 Republic of Korea

## Abstract

Despite efforts to interrogate human genome variation through large-scale databases, systematic preference toward populations of Caucasian descendants has resulted in unintended reduction of power in studying non-Caucasians. Here we report a compilation of coding variants from 1,055 healthy Korean individuals (KOVA; Korean Variant Archive). The samples were sequenced to a mean depth of 75x, yielding 101 singleton variants per individual. Population genetics analysis demonstrates that the Korean population is a distinct ethnic group comparable to other discrete ethnic groups in Africa and Europe, providing a rationale for such independent genomic datasets. Indeed, KOVA conferred 22.8% increased variant filtering power in addition to Exome Aggregation Consortium (ExAC) when used on Korean exomes. Functional assessment of nonsynonymous variant supported the presence of purifying selection in Koreans. Analysis of copy number variants detected 5.2 deletions and 10.3 amplifications per individual with an increased fraction of novel variants among smaller and rarer copy number variable segments. We also report a list of germline variants that are associated with increased tumor susceptibility. This catalog can function as a critical addition to the pre-existing variant databases in pursuing genetic studies of Korean individuals.

## Introduction

The recent population explosion and a limited purifying selection process during recent human evolutionary history caused an over-accumulation of rare variants of varying functionalities in the human genome, creating limitations in pursuing various disease genetic studies^1, 2^. To circumvent such limitations, large-scale databases containing variants from normal healthy populations have been established to provide a ‘healthy genomic profile’, e.g., 1000 Genomes Project (1000GP), Exome Aggregation Consortium (ExAC), and UK10K^3–6^. However, Caucasians comprise the majority of subjects in such databases, leaving ethnic Koreans with a world population of over 70 million underrepresented^7^. Although ~4,300 (7.1%) East Asian samples are included in ExAC, they are mainly from Japan, China, and Southeast Asia. Two Japanese groups recently reported genomic profiles of the Japanese population through 1,070 whole genome sequencing (WGS) and 1,208 whole exome sequencing (WES) data, further characterizing the genetic architecture of the population^8, 9^. The lack of such a Korean database remains as a major obstacle to genetic research and clinical diagnosis on Korean patients with genetic diseases^10^.

Although ambiguous, modern humans (*Homo sapiens sapiens*) are speculated to have first migrated into Northeast Asia approximately 40,000 years ago^11^. There are two proposed hypotheses explaining migration routes to the Northeast Asia: solely through south-to-north migration from Southeast Asia, and a mixture of south-to-north migration and another through Central Asia^12^. Given that southern Chinese and Southeast Asian harbor a relatively heavier enrichment of Denisovan components in their genome than northern Chinese, Northeast Asians seem to have separate origins from the Southeast Asians^13^. This would have also occurred about 40,000 years ago when modern humans first settled in the Korean Peninsula. The first settlers in the Japanese archipelago most likely arrived at Kyushu island, which is across the sea from the Korean Peninsula ~35,000–38,000 years ago^11, 14^. Although there have been continuous migrations and international contacts among the Northeast Asian countries, Korean, Chinese and Japanese populations have remained largely exclusive and existed as genetically distinct populations as indirectly reflected by their distinct languages and cultures^15^. Therefore, elucidating the genetic similarity and differences among the populations in this region will be an invaluable task^15^.

Here we compiled high-quality coding variant data from 1,055 healthy Korean individuals by whole exome sequencing (KOVA; Korean Variant Archive). This variant archive will allow for an enhanced understanding of the Korean genome to assist with research and proper clinical treatment of Korean individuals through genome sequencing, and can advance our understanding of the East Asian genome profile and history.

## Results

### Quality control

To establish a genetic database of healthy Korean individuals, WES data from normal tissues from cancer patients (675 samples; 472 blood and 203 adjacent normal tissues) and blood samples from healthy individuals with no apparent clinical history (380 samples) were collected. Clinical characteristics of the participants are summarized in Supplementary Table S1. After removing duplicated samples and cryptic relatives, 1,055 individuals remained for further analysis. The mean coverage depth of the runs was 75x (Supplementary Table S1). In total, we have identified 293,049 variants after vigorous filtering, which constituted the Korean variant archive (KOVA).

The variants were covered 41x on average and mean genotype quality was above 66 (Supplementary Fig. S1a and b), assuring good variant calling quality. As further quality control steps, we checked transition-to-transversion and hetero-to-homozygosity ratios of the variants (Supplementary Fig. S1c and d and Fig. S2), both of which were consistent with the previous report^16^. Since our data set is a collection from five independent groups with different capture and sequencing platforms, careful exclusion of any systematic bias was necessary. Therefore, we checked for the presence of inter-group biases using several criteria. First, the number of called variants per each individual was approximately ~42 K on average and the distribution was almost similar except for individuals from one group with a slightly lower mean value of ~39 K (Supplementary Fig. S3a), which might be due to using different exome capture kits (Supplementary Table S1). Next, we examined the profile of singleton variants that were seen only once in the set. The number of singletons per each individual was 101 on average and its distribution was stable across different groups (Supplementary Fig. S3b).

### Basic KOVA features and population genetics

**Figure 1:**
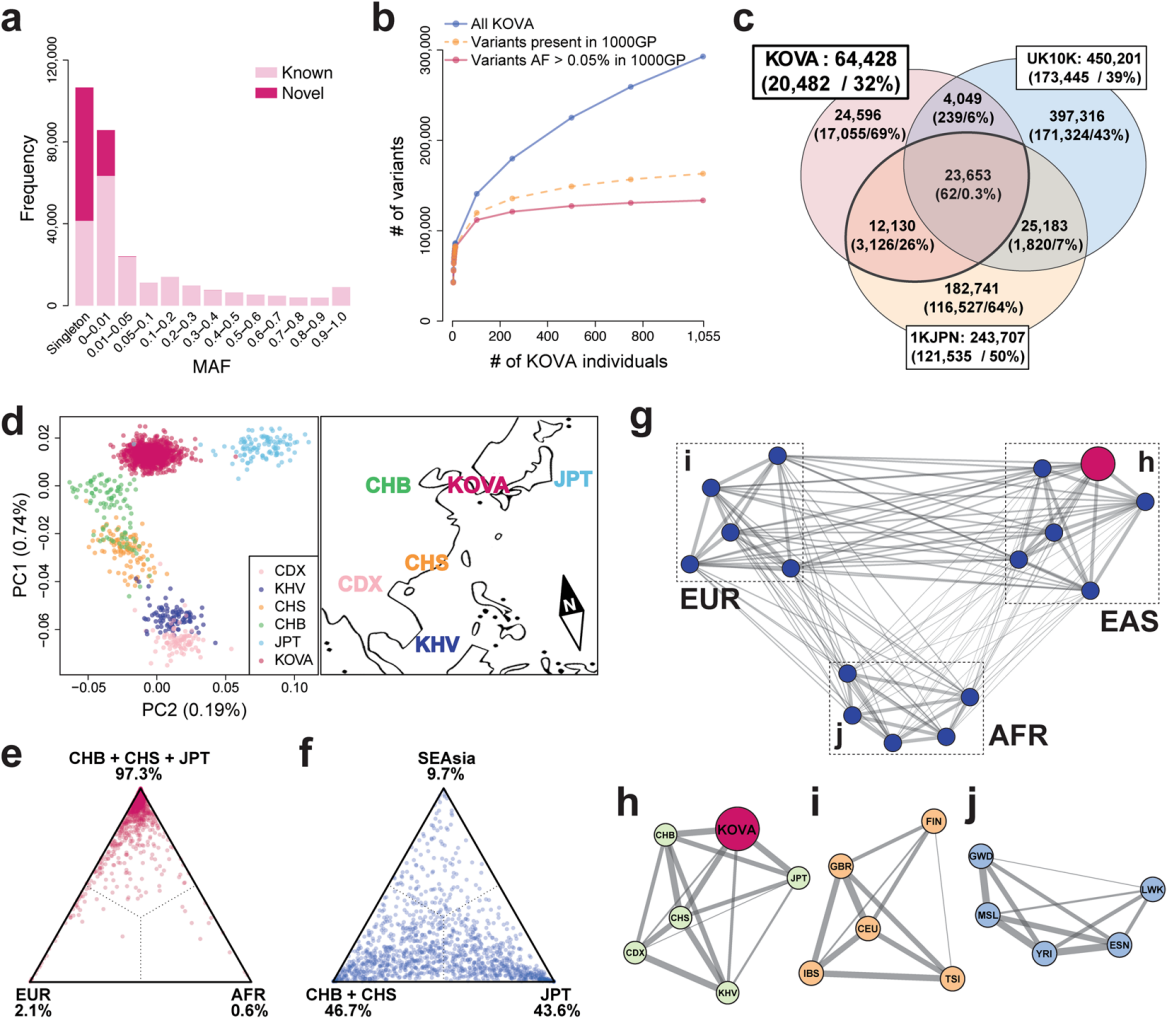
Population profile of KOVA. (**a**) Distribution of variant minor allele frequencies (MAFs). (**b**) Variant increment patterns as the number of the participants increases. (**c**) Venn diagram of coding variant comparisons among KOVA, Japanese population, and UK10K^6, 8^. Numbers and proportion of novel variants (i.e. not in dbSNP build 142) in each area are shown in the parentheses. (**d**) Principal component analysis of KOVA and East Asian populations from 1000 Genomes Project (left panel) and corresponding geographical locations (right panel). The map image was modified from Openclipart with permission. (**e** and **f**) Gene-level *F*_*ST*_ between KOVA and (**e**) East Asian, European, and African populations, and (**f**) Chinese, Japanese, and Southeast Asian populations. Each dot indicates a gene (see methods) and percentage values beneath population names denote proportion of dots that fell in each sector. (**g**) Network plot depicting pairwise fixation index (*F*_*ST*_) of multiple population groups including KOVA, which is represented as a red node. Thicker line indicates smaller *F*_*ST*_, indicating closer relationship. Positions of the nodes are arbitrarily arranged to roughly reflect the geographical location. Each subpopulation of (**h**) EAS including KOVA, (**i**) EUR, and (**j**) AFR was drawn separately. 1000GP; 1000 Genomes Project, AFR; African excluding Americans of African Ancestry in southwestern USA and African Caribbeans in Barbados, CDX; Chinese Dai in Xishuangbanna, China, CEU; Utah Residents (CEPH) with Northern and Western Ancestry, CHB; Han Chinese in Bejing, China, CHS; Southern Han Chinese, EAS; East Asian, ESN; Esan in Nigeria, EUR; European, FIN; Finnish in Finland, GBR; British in England and Scotland, GWD; Gambian in Western Divisions in the Gambia, IBS; Iberian Population in Spain, JPT; Japanese in Tokyo, KHV; Kinh in Ho Chi Minh City, Vietnam, LWK; Luhya in Webuye, Kenya, MSL; Mende in Sierra Leone, SEAsia; CDX and KHV, TSI; Toscani in Italia, YRI; Yoruba in Ibadan, Nigeria.

Identification of Korean-specific variations and cataloging their frequencies are among the main purposes of the study. Comparison with the dbSNP database (build 147) showed that 205,002 (70.0%) variants were known and 88,047 (30.0%) variants were novel. Distribution of minor allele frequency (MAF) showed enrichment of novel variants in rare variants (45.6% of MAF ≦ 0.01) and the highest in the singletons (61.2%), consistent with previous reports (Fig. 1a, Supplementary Table S2)^5, 6^. Common variants of MAF > 0.05 were mostly known variants reported in dbSNP (99.8%). Simulation analysis demonstrates that the common variants reported in the 1000GP are rapidly saturated as the number of KOVA samples increases, suggesting that we are capturing almost all Korean common coding variants using this set, while rare or singleton variants accumulate with minimal overlaps as individual number increases (Fig. 1b). Therefore, KOVA can confer additional contributions to variant filtering in addition to the pre-existing databases when performing human genetics studies as confirmed by a simulation study using an independent Korean WES data set (22.8% additional reduction conferred to the ExAC-filtered variants; Supplementary Fig. S4).

To better understand the relationship of variant profiles between Korean and other populations, we compared the KOVA variants with those from other whole exome or genome sequencing based population studies. Comparison with the UK10K data would delineate the difference between Caucasian and Asian populations, and comparison with the Japanese whole genome sequencing (1KJPN) data is expected to contrast the subtle difference between Korean and Japanese populations^6, 8^. Only 36.7% of KOVA variants (23,653/64,428) were commonly found in all three populations, indicating substantial differences by ethnic background (Fig. 1c and Supplementary Fig. S5). From this comparison, the number of UK10K-specific variants were the largest with 397,316 variants, which is expected given its large cohort size. As we applied strict variant filtering strategy, we noted that the number of KOVA-specific variants is markedly lower than that of the Japanese population. Principal component analysis (PCA) revealed that KOVA co-clustered with East Asians (Supplementary Fig. S6a) and located between Northern Chinese and Japanese (Fig. 1d). The PCA plot was in good agreement with the geographical locations of corresponding ethnic groups (Fig. 1d). As expected, fixation index (*F*_*ST*_ *)* analysis, a parameter of population differentiation, revealed the closest relationship as Korean to Japanese and Chinese over African, European and Southeast Asian (Fig. 1e and f and Supplementary Fig. S6b and c). Having large genome-wide variant information, we asked how distinct is the Korean population compared to East Asian neighbors and to among the ethnic groups in African or European continents. Calculating *F*_*ST*_ among multiple population groups from 1000GP and KOVA reveals a close genetic relationship within each super-population and that KOVA is closely related to EAS in the 1000GP as expected (Fig. 1g). One of the critical questions that can be addressed by this study is whether Korean population is genetically distinct from its neighboring populations, which will provide the rationale for constructing its own variant database. In this respect, we noted that the degree of closeness between KOVA and other East Asian populations, as scored by weighted *F*_*ST*_, is comparable or larger than those between African or European populations (Fig. 1g–j), demonstrating that the Korean population is a distinct ethnic group among EAS as those from other continents.

### Functional analysis of coding variants

**Figure 2:**
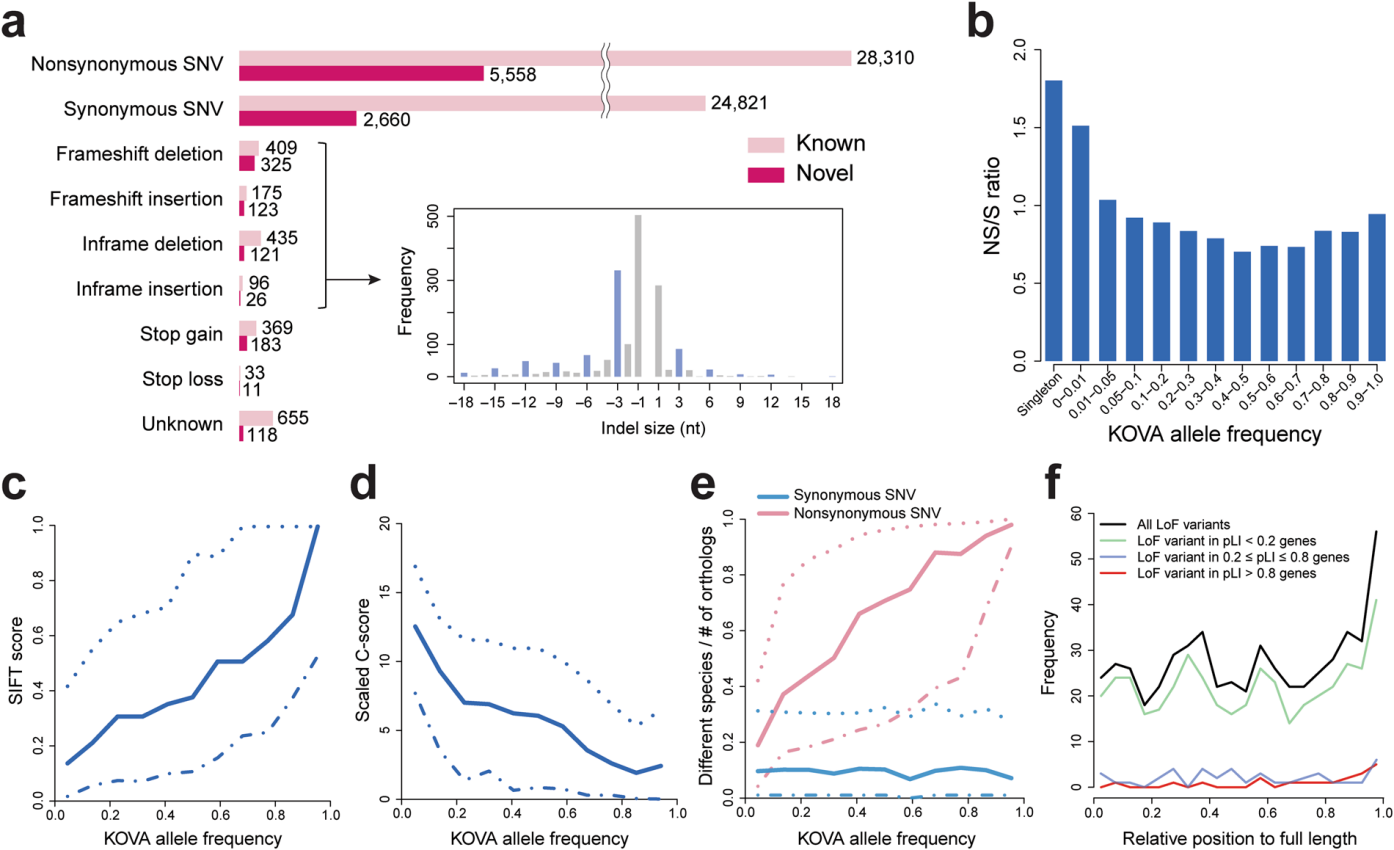
Functional analysis of KOVA coding variants. (**a**) Numbers of novel and known variants categorized by function. The overlaid plot shows size distribution of indels, with the blue bar indicating multiples of three bases. (**b**) Nonsynonymous to synonymous SNV (NS/S) ratio by variant allele frequencies. (**c**) SIFT score and (**d**) Scaled C-score (CADD) by allele frequencies. (**e**) Degree of amino acid conservation of variant residues by allele frequencies. Fraction of species numbers with different amino acid on orthologous proteins compared to human orthologs. (**f**) Relative position of loss-of-function (LoF) variants on protein. Solid, dotted, and dash-dot lines in c-e indicate median, upper, and lower quantiles, respectively.

Next, we analyzed the functional impact of 64,428 coding variants in KOVA (Table 1). The portion of novel coding variants was 14.2% and most of the exonic variants were SNVs (95.2%). Short insertions and deletions (indels) are predominantly smaller than seven bases (93.8%), and coding indels are enriched in multiples of three bases, consistent with previous findings (Fig. 2a and Table 1). The novel-to-known ratio is relatively high in functionally significant variants such as frame-shift indels, stop gains and stop losses presumably due to purifying selection.

**Table 1:** Summary of Exonic Variants in KOVA.

Types	Total	Known (dbSNP147)	Novel
Nonsynonymous SNV	33,868	28,310	5,558
Synonymous SNV	27,481	24,821	2,660
Frameshift deletion	734	409	325
Frameshift insertion	298	175	123
Inframe deletion	556	435	121
Inframe insertion	122	96	26
Stop gain	552	369	183
Stop loss	44	33	11
Unknown	773	655	118
Total Coding	64,428	55,303	9,125

The nonsynonymous-to-synonymous SNVs (N/S) ratio was relatively high in rare variants (Fig. 2b). The nonsynonymous variants tend to display more damaging or pathogenic scores as variants become rarer in the population (i.e., reduced SIFT score, increased PolyPhen-2, scaled C-score (CADD score) and PhyloP scores) (Fig. 2c and d and Supplementary Fig. S7)^17–20^, again implying increased variant functionality as they become relatively rare. One important parameter of nonsynonymous variant functionality is how an amino acid residue is conserved throughout evolution. Therefore, we counted the ratio of vertebrate species with different amino acids on orthologous proteins from human as a measurement of evolutionary conservation. The ratio increased (i.e., weaker conservation) for nonsynonymous variant residues as allele frequency increases, whereas the ratio remained consistently low (i.e., strong conservation) for synonymous ones (Fig. 2e). This result suggests that nonsynonymous variants, especially common ones, tend to occur in less well conserved residues, mostly escaped from functional restrictions conferred by amino acid changes. The mean of 117 heterozygous loss-of-function (LoF) variants were detected per individual. A majority of LoF variants are rare (85.3% of all LoF variants are MAF < 1%), and more than half of LoF are singletons (55.8%). Consistent with the observation from a nonsynonymous variant conservation pattern, the relative positions of stop gain variants on proteins were biased to the C-terminal end (Fig. 2f) and most of the stop gain variants were found in genes with tolerable pLI (probability of being LoF intolerant) values (<0.2)^5^. Although the participants of the study are healthy, we compared the KOVA variants to the ClinVar list to test whether there are any carriers of reported pathogenic variants (Supplementary Fig. S8). A number of “pathogenic” variants that reach high allele frequencies were detected, supporting the rising concern that a certain portion of previously-tagged pathogenic variants may not be truly pathogenic (26 variants are MAF > 10% in both KOVA and ExAC, Supplementary Fig. S8).

### Copy number variations in KOVA

**Figure 3:**
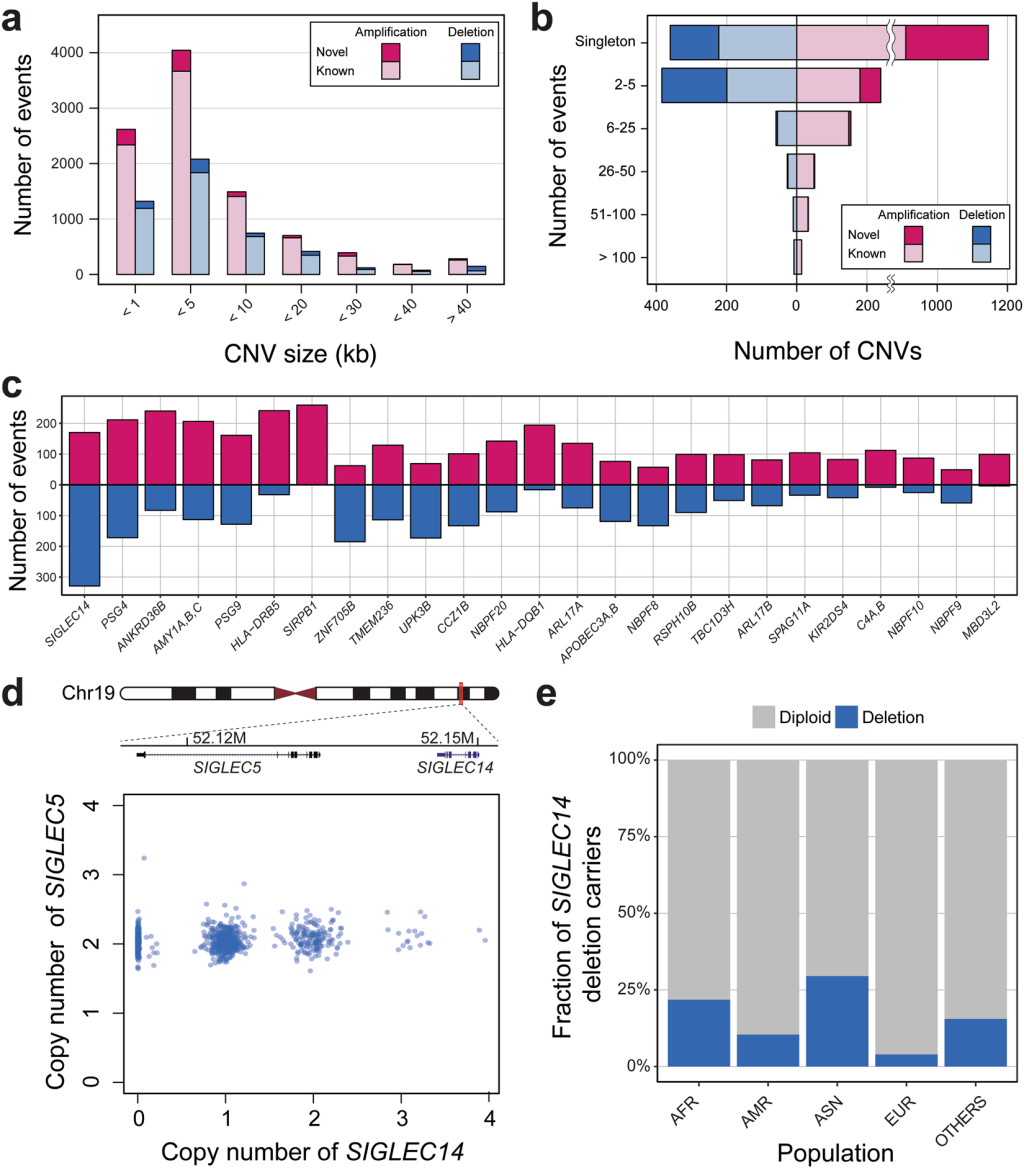
Copy number variations in KOVA. (**a**) Distribution of KOVA CNV sizes. (**b**) Frequency of CNVs by number of events in KOVA. (**c**) Highly polymorphic copy number genes in KOVA. Genes are sorted by frequency. (**d**) Copy number genotype profiles of *SIGLEC14* and *SIGLEC5*. (**e**) Frequency of *SIGLEC14* deletion allele in worldwide populations from DGV. AFR: African, AMR: Mexican, native American, North American, and South American, ASN: Asian, EUR: European.

Although WES is not designed for copy number variation (CNV) detection, the large sample size and recent improvements of bioinformatics tools allow stable CNV detection and analysis^5^. After the initial QC filtering steps, 944 samples remained and were used for CNV analysis. Among the 944 Korean individuals, we found a total of 14,600 putative CNV segments. The average number of CNVs per individual was 10.3 and 5.2 for the amplifications and deletions, respectively (Supplementary Table S3). Most of the called CNV segments were shorter than 10 kb (i.e. segments covering two or three exons), as larger CNVs tend to pose higher odds of conferring pathogenicity (Fig. 3a). We compared our CNV segments with control list of CNVs from the database of genomic variants (DGV) (see Materials and Methods). About 10% of all KOVA CNV segments were not found in DGV and were called as novel. These novel CNV calls tend to be smaller in size (78.1% smaller than 10 kb) and found in rare frequencies (Fig. 3a and b). Chromosome 19 was known to display the highest gene density among the human chromosomes^21^, and contained the highest numbers of CNV calls after adjusting for size, consistent with other WES-based CNV studies (Supplementary Fig. S9)^22^. To find highly copy number polymorphic genes in the Korean population, we counted the frequency of copy number changes by sorting genes by sum of amplification and deletion frequencies (Fig. 3c). The list includes well-known polymorphic loci such as amylase and *HLA* genes, and *SIGLEC14* was the most highly copy number polymorphic locus while *SIGLEC5*, which is located just upstream of *SIGLEC14* is mostly copy-neutral (Fig. 3d and Supplementary Figs S10 and S11). A previous study reported that deletion polymorphism of *SIGLEC14* is higher in Asians compared with Africans and Europeans^23^. We also observed this Asian-specific high frequency of *SIGLEC14* deletion in DGV, although its functional implication remains elusive (Fig. 3e). Finally, a subset of KOVA subjects (n = 208) were also analyzed by SNP arrays and their CNV profiles are well-correlated with WES-based KOVA profiles (Correlation = 0.43, Supplementary Fig. S12).

### Potential role of rare germline variants on tumor susceptibility

**Figure 4:**
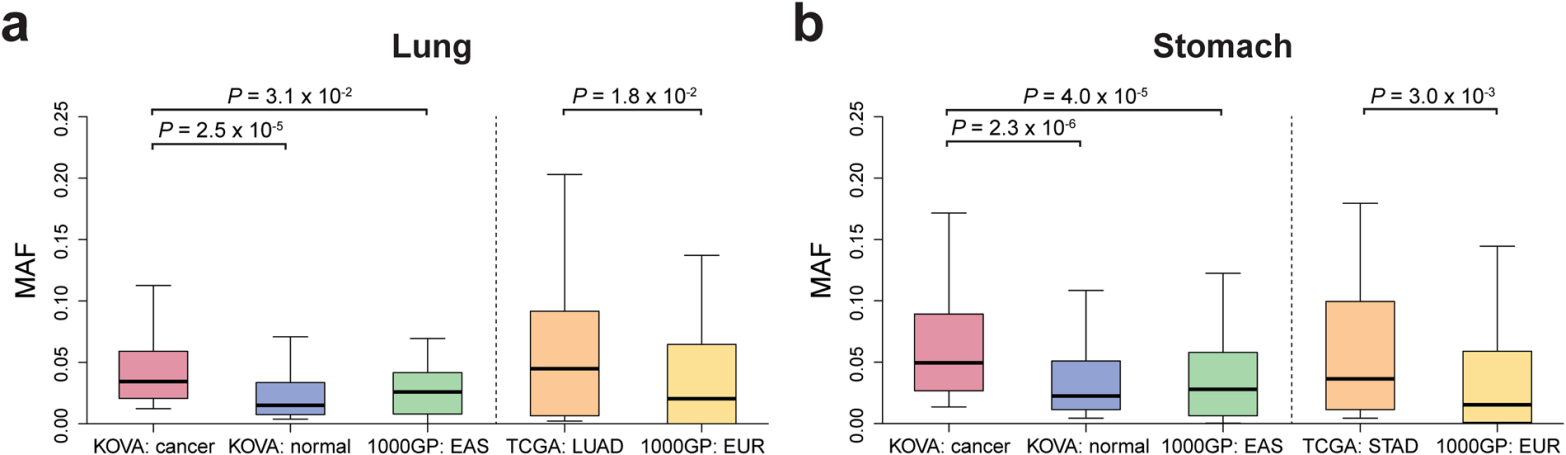
Cancer susceptibility variant distributions in KOVA. Potentially deleterious SNV MAFs extracted from (**a**) lung adenocarcinoma and (**b**) stomach adenocarcinoma tumor-paired normal sets or other public databases were plotted. LUAD: lung adenocarcinoma; STAD: stomach adenocarcinoma.

Having tumor-paired normal samples in our cohort provided an opportunity to explore the potential role of rare germline variants in cancer development. After selecting 54 and 72 rare variants for lung and stomach cancers (Supplementary Tables S4 and S5), that were enriched in “tumor-paired normals” compared to “healthy normals” (see Materials and Methods), we observed a similar difference of allele frequencies between the Cancer Genome Atlas (TCGA) and 1000GP normal datasets (*P* = 0.018 and *P* = 0.003 for lung and stomach adenocarcinomas, respectively; Fig. 4). Our results indicate that certain SNVs in our tumor-derived cohorts may potentially function as predisposing factors for tumorigenesis.

## Discussion

To our knowledge, a WES-based genomic variant catalog from Korean individuals of this size has never been previously reported. We have demonstrated the rationale of constructing an independent Korean genomic database by showing that genetic distances between KOVA and other East Asian ethnic groups are comparable or even farther than those between the ethnic groups of Africa or Europe (Fig. 1g–j). In East Asia, one of the most genetically similar ethnic groups to Koreans is Japanese, which is well supported by historic evidence that people have colonized the Japanese archipelago through the Korean Peninsula about 40,000 years ago. The high-quality coding variants that are predicted to change protein sequences followed signatures of purifying selection. Although we need to further investigate functional implications and multi-ethnic comparative profiling of Korean copy number variations, we called 14,600 CNVs and demonstrate that they also followed the restrictions posed by purifying selection. We also propose a group of rare functional variants that may regulate cancer susceptibility and validated their consistent behaviors using European-based TCGA and 1000GP databases. Further validation of these variants is required with larger independent cohorts and performing functional analyses.

There are limitations in this study, one of which is our strategy of applying stringent variant filtering criteria, which resulted in calling fewer variants compared to others. As the number of rare variants continued to increase as we added more samples (Fig. 1b), we are still limited in covering rare variants using this cohort size. Nevertheless, this study cataloged the largest healthy Korean cohorts and we found that most of the common coding variants were well covered by this set.

WES- or WGS-based sequencing efforts have become more commonplace over recent years and will continue to do so in the near future, thus we anticipate that expanding KOVA with new participants will ensure that the archive remains a valuable database for pursuing disease-based, population or evolutionary genetic studies of Korean individuals.

## Methods

### Cohorts and sample preparation

We collected WES data of Korean individuals from five independent research groups. All sequencing data were obtained from normal tissues or blood samples following standard protocols (Supplementary Table S1). This project was performed with approval of the Institutional Review Board of each group (Seoul National University, Ewha Womans University, Asan Medical Center, and Samsung Medical Center), in which all donors provided written informed consent. All the experiments were performed in de-identified status and in accordance with relevant guidelines and regulations.

### Variant calling and filtering

The raw sequencing data were analyzed with in-house pipeline (Supplementary Fig. S13) to combine data from different exome capture platforms. Briefly, BWA (version 0.7.5a) was used to map short reads to hg19. GATK (version 2.4–7) was used for local realignment and recalibration after duplicate marking with Picard (version 1.93)^24^. GATK UnifiedGenotyper was used to call variants across all samples simultaneously in the multi-sample calling mode, which allowed us to distinguish whether no variant calls indicated homozygous reference or missing calls due to low coverage. The quality score was further recalibrated using GATK’s VQSR model. To obtain a reliable list of variants suitable for population genetics studies, we applied extensive filtering steps as per the following: The minimum genotype quality and depth of coverage were set to 30 and 10, respectively. Then variants with missing genotypes in more than 30% of all individuals were excluded from further analyses. We also removed variants that violate Hardy-Weinberg equilibrium on allelic frequency (*P* < 10^−6^).

### Power simulation of KOVA

To simulate variant increment pattern as the number of KOVA individuals increases, we randomly selected 1, 2, 3, 4, 5, 6, 7, 8, 9, 10, 100, 250, 500 and 750 samples from KOVA to count variants of MAF > 0.05% in 1000GP and novel variants.

### Principal component and fixation index (F_ST_) analyses

Throughout the study, “African” indicates populations with a super-population code of AFR in the 1000GP phase 3, excluding ASW (Americans of African Ancestry in SW USA) and ACB (African Caribbeans in Barbados), and “South East Asian” includes CDX (Chinese Dai in Xishuangbanna, China) and KHV (Kinh in Ho Chi Minh City, Vietnam)^4^. Variants intersecting 1000GP phase 3 and KOVA were merged to a VCF file using vcftools (version 0.1.15)^25^. For comparison of KOVA and EAS (East Asian) in the 1000GP, merged VCF file was organized by sample names using ‘bcftools subset’ (version 1.3, https://github.com/samtools/BCFtools). These VCF files were independently filtered using the ‘bcftools view’ to retrieve common (MAF > 5%) variants and then used as inputs for principal component analysis (PCA) and *F*_*ST*_. PCA was performed with SNPRelate R package (version 1.4.2)^26^. *F*_*ST*_ was estimated with vcftools using–weir-fst-pop option^25, 27^. Variant level analysis was performed with a 0.5 million bp window size (option:–fst-window-size 500000) and 0.5 million bp step size (option:–fst-window-step 500000). Windows containing less than three variants were excluded from the subsequent analyses. For gene level analysis, we estimated *F*_*ST*_ for individual variants and then assigned them to each gene according to intervals annotated in the GTF file of GENCODE 19^28^. Genes with over ten variants were included in the analysis. *F*_*ST*_ values were converted to positions representing relative distances to each ethnic group and visualized on a triangle plot. For network analysis using *F*_*ST*_ values, genome-wide weighted *F*_*ST*_ values were used as inputs to Cytoscape (version 3.4.0)^29^.

### Functional annotation of nonsynonymous variants

Functional annotation was performed by ANNOVAR (version 2014-11-13) with databases summarized in Supplementary Table S6 ^30^. Multiple alignment of orthologous protein was downloaded from the UCSC genome browser database.

### Copy number variation analysis

We called CNVs with CODEX software using default settings^31^. To adjust for possible variations derived from different exome capture kits, we applied the algorithm to each group separately and then combined the results. Known CNVs were downloaded from the DGV (Database of genomic variants, http://dgv.tcag.ca) and calls from 2009 and onwards that were generated using WGS or SNP array platforms were selected for subsequent analyses. The overlap between the DGV variants and KOVA CNV segments were calculated with bedtools using −r 0.5 option (i.e., 50% overlap)^32^. Notable CNV segments were manually checked using the Integrative Genomics Viewer (IGV). ExAC copy number data were downloaded from the ftp site (release 0.3.1, ftp://ftp.broadinstitute.org)^5, 22, 33^. DGV dataset ID:gssvL59302 was used for global *SIGLEC14* copy number profile.

In order to validate the KOVA CNV from an independent platform, we performed CytoScan HD array scanning on 208 samples. Briefly, raw CEL files were processed by apt-copynumber-cyto (1.18.2) from Affymetrix Power Tools (APT). Details are described in APT manual (http://media.affymetrix.com/support/developer/powertools/changelog/apt-copynumber-cyto.html). Then the KOVA absolute copy number and array signal intensity were compared after exclusion of non-overlapping probes.

### Assessing roles of rare germline variants on tumor susceptibility

The SNVs from the tumor-paired normal individuals (“tumor-paired normal”, 364 and 76 samples for lung and stomach adenocarcinoma, respectively) and healthy normal individuals (“healthy normal”, 134 samples) were filtered from a single collective VCF file using the VCFtools–keep option^25^. MAF of the SNVs from tumor-paired normals was compared to that of the SNVs from healthy normals. Protein-altering SNVs that exhibited MAF < 0.01 and 1.5 fold greater MAF in the tumor-paired normals than the heathy normals were selected for further analysis. These SNVs were annotated using ANNOVAR for four functional prediction parameters (SIFT, PolyPhen-2, MutationAssessor and GERP++)^30, 34^. The SNVs that were predicted to be deleterious in two out of the four prediction tools were selected as potentially interesting candidates of tumor susceptibility.

For a validation test, the tumor-paired normal data of the lung (LUAD, n = 229) and stomach (STAD, n = 137) adenocarcinomas from TCGA was obtained after excluding non-whites using the analysis pipeline outlined in Supplementary Fig. S13. The SNV data from the 504 East Asian (EAS) and 503 European (EUR) individuals in 1000GP were obtained as independent healthy normal cohorts using VCFtools to compare with tumor-paired normals in KOVA and TCGA, respectively^3, 4, 25^. The selected SNVs from the discovery phase was compared with those of EAS individuals in 1000GP and between those of TCGA and EUR individuals in 1000GP, and the significance was assessed using Wilcoxon’s rank sum test.

## Electronic supplementary material

Supplementary file

## References

[1] Fu W (2013). Analysis of 6,515 exomes reveals the recent origin of most human protein-coding variants. Nature.

[2] Tennessen JA (2012). Evolution and functional impact of rare coding variation from deep sequencing of human exomes. Science.

[3] 1000 Genomes Project Consortium *et al*. An integrated map of genetic variation from 1,092 human genomes. *Nature* **491**, 56–65 (2012). 10.1038/nature11632 PMC3498066 23128226

[4] 1000 Genomes Project Consortium *et al*. A global reference for human genetic variation. *Nature* **526**, 68–74 (2015). 10.1038/nature15393 PMC4750478 26432245

[5] Lek M (2016). Analysis of protein-coding genetic variation in 60,706 humans. Nature.

[6] Huang J (2015). The UK10K project identifies rare variants in health and disease. Nature.

[7] Population Division, Department of Economic and Social Affairs, United Nations. *World Population Prospects: The 2015 Revision, Key Findings and Advance Tables*. Available from: https://esa.un.org/unpd/wpp/publications/files/key_findings_wpp_2015.pdf (2015).

[8] Nagasaki M (2015). Rare variant discovery by deep whole-genome sequencing of 1,070 Japanese individuals. Nat Commun.

[9] Higasa K (2016). Human genetic variation database, a reference database of genetic variations in the Japanese population. J Hum Genet.

[10] Petrovski S, Goldstein DB (2016). Unequal representation of genetic variation across ancestry groups creates healthcare inequality in the application of precision medicine. Genome Biol.

[11] Stanyon R, Sazzini M, Luiselli D (2009). Timing the first human migration into eastern Asia. J. Biol..

[12] Jin H-J, Tyler-Smith C, Kim W (2009). The peopling of Korea revealed by analyses of mitochondrial DNA and Y-chromosomal markers. PLoS ONE.

[13] Skoglund P, Jakobsson M (2011). Archaic human ancestry in East Asia. Proc. Natl. Acad. Sci. USA.

[14] Takashi T (2012). MIS3 edge-ground axes and the arrival of the first Homo sapiens in the Japanese archipelago. Quat Int.

[15] Tian C (2008). Analysis of East Asia genetic substructure using genome-wide SNP arrays. PLoS ONE.

[16] Wang J, Raskin L, Samuels DC, Shyr Y, Guo Y (2015). Genome measures used for quality control are dependent on gene function and ancestry. Bioinformatics.

[17] Adzhubei IA (2010). A method and server for predicting damaging missense mutations. Nat. Methods.

[18] Kircher M (2014). A general framework for estimating the relative pathogenicity of human genetic variants. Nat. Genet..

[19] Kumar P, Henikoff S, Ng PC (2009). Predicting the effects of coding non-synonymous variants on protein function using the SIFT algorithm. Nat Protoc.

[20] Cooper GM (2005). Distribution and intensity of constraint in mammalian genomic sequence. Genome Res.

[21] Grimwood J (2004). The DNA sequence and biology of human chromosome 19. Nature.

[22] Ruderfer DM (2016). Patterns of genic intolerance of rare copy number variation in 59,898 human exomes. Nat. Genet..

[23] Yamanaka M, Kato Y, Angata T, Narimatsu H (2009). Deletion polymorphism of SIGLEC14 and its functional implications. Glycobiology.

[24] McKenna A (2010). The Genome Analysis Toolkit: a MapReduce framework for analyzing next-generation DNA sequencing data. Genome Res.

[25] Danecek P (2011). The variant call format and VCFtools. Bioinformatics.

[26] Zheng X (2012). A high-performance computing toolset for relatedness and principal component analysis of SNP data. Bioinformatics.

[27] Weir BS, Cockerham CC (1984). Estimating F-statistics for the analysis of population structure. evolution.

[28] Harrow J (2012). GENCODE: the reference human genome annotation for The ENCODE Project. Genome Res.

[29] Shannon P (2003). Cytoscape: a software environment for integrated models of biomolecular interaction networks. Genome Res.

[30] Wang K, Li M, Hakonarson H (2010). ANNOVAR: functional annotation of genetic variants from high-throughput sequencing data. Nucleic Acids Res..

[31] Jiang Y, Oldridge DA, Diskin SJ, Zhang NR (2015). CODEX: a normalization and copy number variation detection method for whole exome sequencing. Nucleic Acids Res..

[32] Quinlan AR, Hall IM (2010). BEDTools: a flexible suite of utilities for comparing genomic features. Bioinformatics.

[33] Thorvaldsdóttir H, Robinson JT, Mesirov JP (2013). Integrative Genomics Viewer (IGV): high-performance genomics data visualization and exploration. Brief Bioinformatics.

[34] Liu X, Jian X, Boerwinkle E (2013). dbNSFP v2.0: a database of human non-synonymous SNVs and their functional predictions and annotations. Hum Mutat.

