# Supplementary material for: Korean Variant Archive (KOVA): a reference database of genetic variations in the Korean population
Source: Sci Rep. 2017 Jun 27;7:4287. doi: 10.1038/s41598-017-04642-4 (PMC5487339; doi:10.1038/s41598-017-04642-4)
Supplement: Supplementary file 1 — Supplementary file [file 41598_2017_4642_MOESM1_ESM.pdf]

SUPPLEMENTARY INFORMATION

Korean Variant Archive (KOVA): a reference database of genetic variations in the Korean population

Sangmoon Lee[1,14], Jihae Seo[2,14], Jinman Park[3,4,14], Jae-Yong Nam[5,6,14], Ahyoung Choi[2,7], Jason S Ignatius[8], Robert D Bjornson[9], Jong-Hee Chae[10], In-Jin Jang[11], Sanghyuk Lee[2,7], Woong-Yang Park[5,6,12], Daehyun Baek[3,4,13,*], and Murim Choi[1,*]

[1]Department of Biomedical Sciences, Seoul National University College of Medicine, Seoul 03080, Republic of Korea

[2]Ewha Research Center for Systems Biology (ERCSB), Ewha Womans University, Seoul 03760, Republic of Korea

[3]Center for RNA Research, Institute for Basic Science, Seoul 08826, Republic of Korea.

[4]School of Biological Sciences, Seoul National University, Seoul 08826, Republic of Korea.

[5]Samsung Genome Institute, Samsung Medical Center, Seoul 06351, Republic of Korea

[6]Department of Health Sciences and Technology, Samsung Advanced Institute of Science and Heath Technology, Sungkyunkwan University, Seoul 06351, Republic of Korea

[7]Department of Bio-Information Science, Ewha Womans University, Seoul 03760, Republic of Korea

[8]Yale Center for Research Computing, Yale University, New Haven, CT 06511, USA.

[9]Department of Computer Science and Yale Center for Research Computing, Yale University, New Haven, CT 06511, USA.

[10]Department of Pediatrics, Seoul National University Children's Hospital, Seoul National University College of Medicine, Seoul 03080, Republic of Korea.

[11]Department of Clinical Pharmacology and Therapeutics, Seoul National University College of Medicine and Seoul National University Hospital, Seoul 03080, Republic of Korea

[12]Department of Molecular Cell Biology, Sungkyunkwan University School of Medicine, Suwon 16419, Republic of Korea

[13]Bioinformatics Institute, Seoul National University, Seoul 08826, Republic of Korea.

[14]These authors contributed equally to this work

[*]These authors contributed equally to this work

Correspondence to:

Daehyun Baek, Ph.D.

School of Biological Sciences, Seoul National University, 1 Gwanak-ro, Gwanak-gu, Seoul 08826, Republic of Korea.

Tel: +82-2-880-4264

E-mail: baek@snu.ac.kr

or

Murim Choi, Ph.D.

Department of Biomedical Sciences, Seoul National University College of Medicine, 103 Daehak-ro, Jongno-gu, Seoul 03080, Republic of Korea

Tel: +82-2-740-8912

Fax: +82-2-3673-2167

E-mail: murimchoi@snu.ac.kr

Running Title: Profile of coding variants from 1,055 Korean individuals

Supplementary Methods

Power simulation of KOVA

We also simulated power of the KOVA database in analyzing actual Korean exome data. WES from 16 Korean patients with congenital neurologic diseases were processed through the same pipeline as KOVA, and then the variants reported in ExAC were filtered out. The number of variants remaining after removing variants from randomly selected sample sets from KOVA were counted.

Relatedness among individuals

Relatedness among individuals was checked by vcftools (version 0.1.15) with --relatedness option.[1,2] Pairs of samples with the output value over 0.1 were regarded as related and excluded.

ClinVar analysis

For ClinVar analysis, variants annotated as pathogenic in clinvar_20150629 database were used for further analysis.

References

1. Yang, L. *et al.* Analyzing Somatic Genome Rearrangements in Human Cancers by Using Whole-Exome Sequencing. *Am. J. Hum. Genet.* **98,** 843–856 (2016).
2. Danecek, P. *et al.* The variant call format and VCFtools. *Bioinformatics* **27,** 2156–2158 (2011).

Supplementary Figures

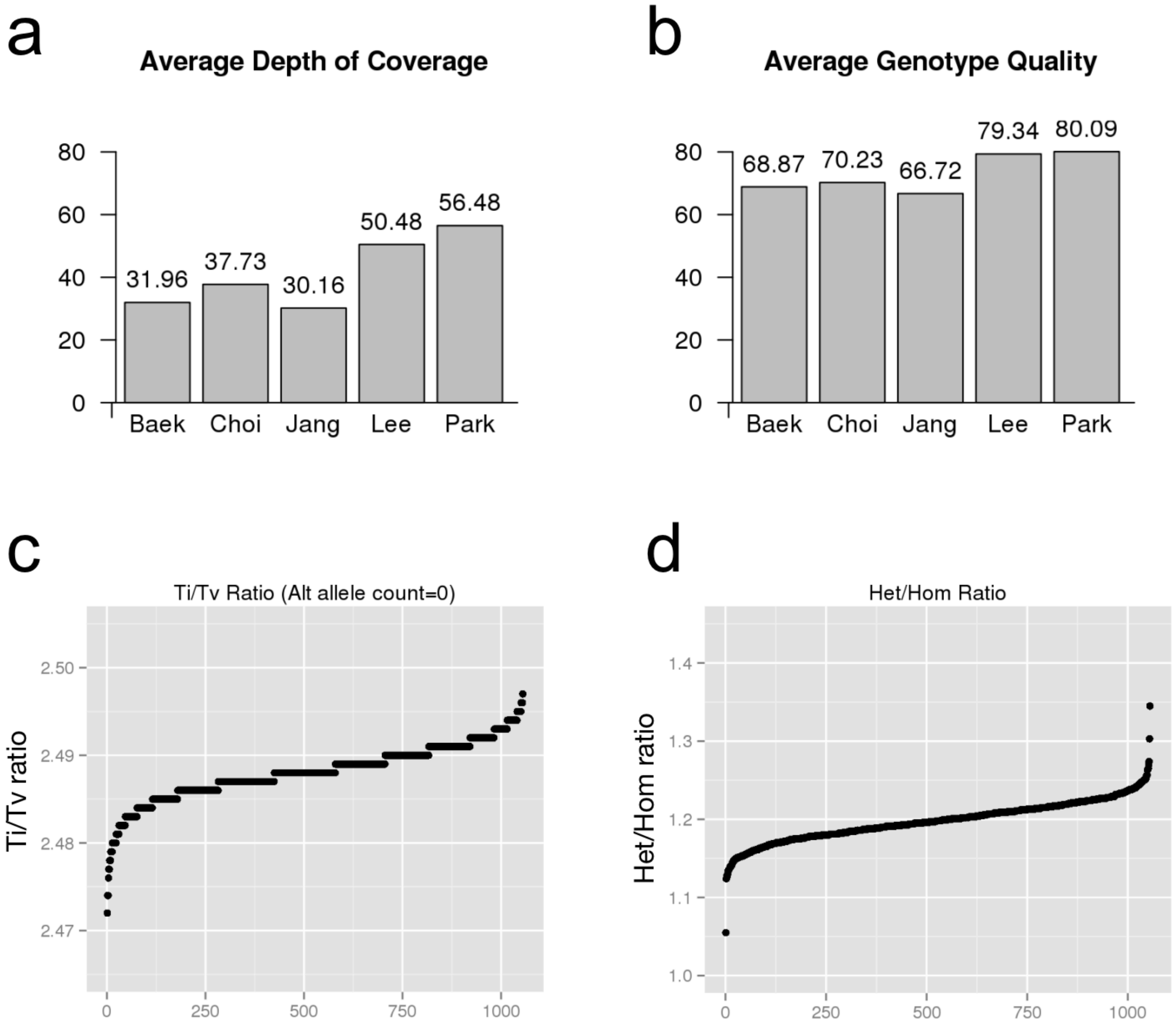

Supplementary Figure S1. Quality control of KOVA. (**a**) Average depth of coverage of variants. (**b**) Average genotype quality. (**c**) Transition/transversion ratio of variants. (**d**) Heterozygous/homozygous ratio of variants.

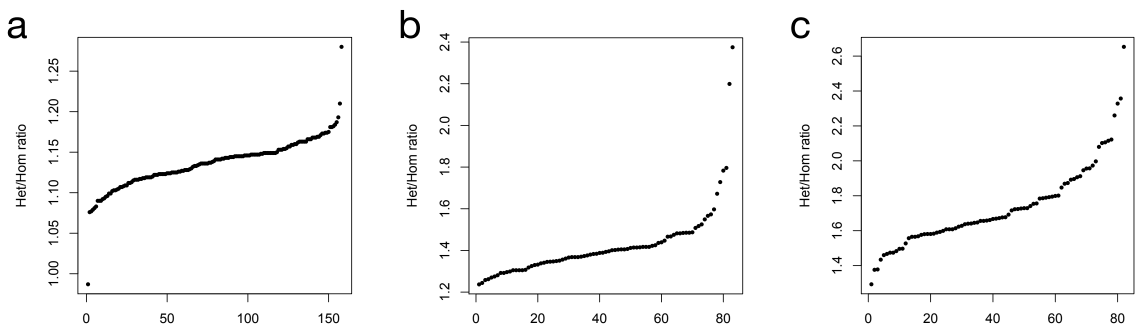

Supplementary Figure S2. Hetero-to-homozygosity ratios of 1000 Genomes Project data analyzed by KOVA pipeline. (**a**) East Asians (n = 158, median = 1,14) (**b**) European (n = 83, median = 1.39) (**c**) African (n = 82, median = 1.67)

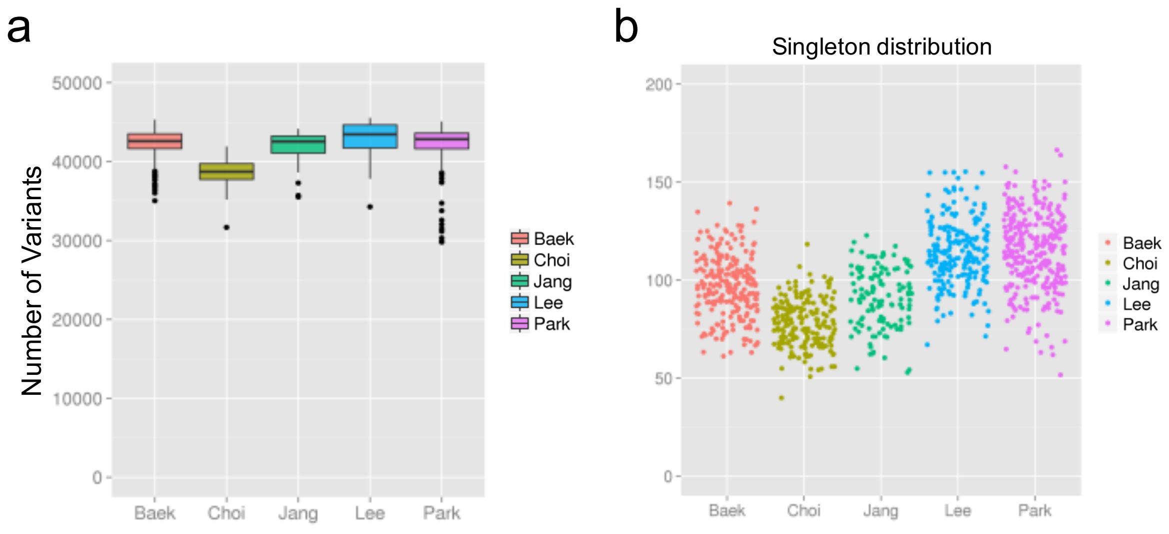

Supplementary Figure S3. Sample-wise number of (**a**) total variants and (**b**) singleton variants.

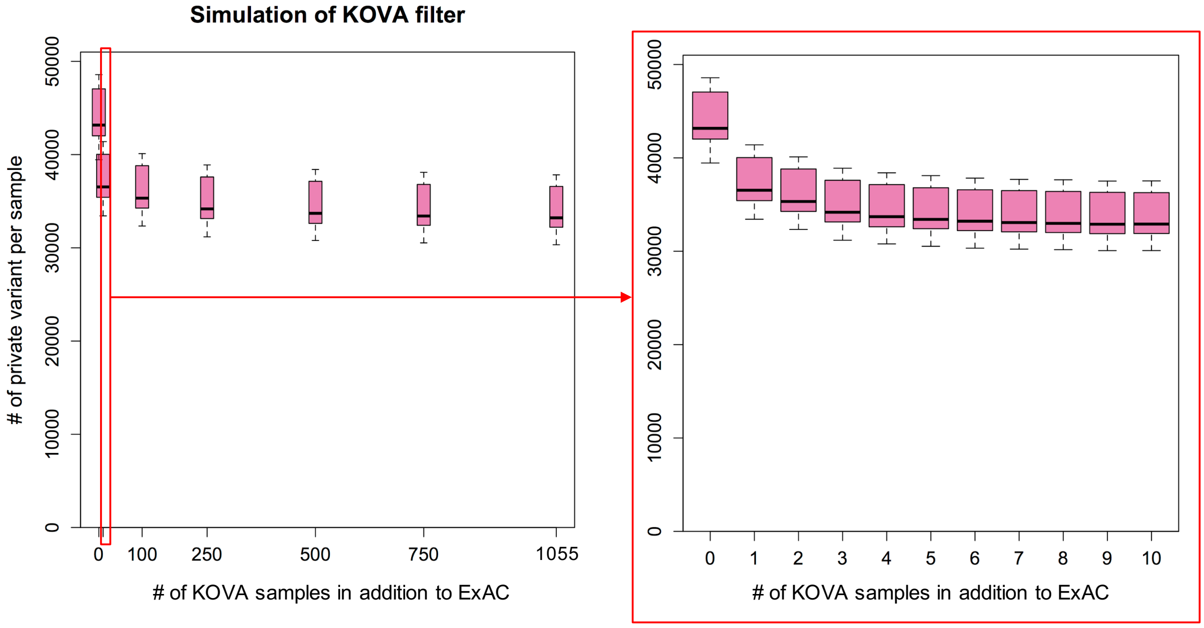

Supplementary Figure S4. Simulation of increasing number of KOVA samples as a filtering option additional to ExAC for independent Korean cohort. Sample number of cohort is 16.

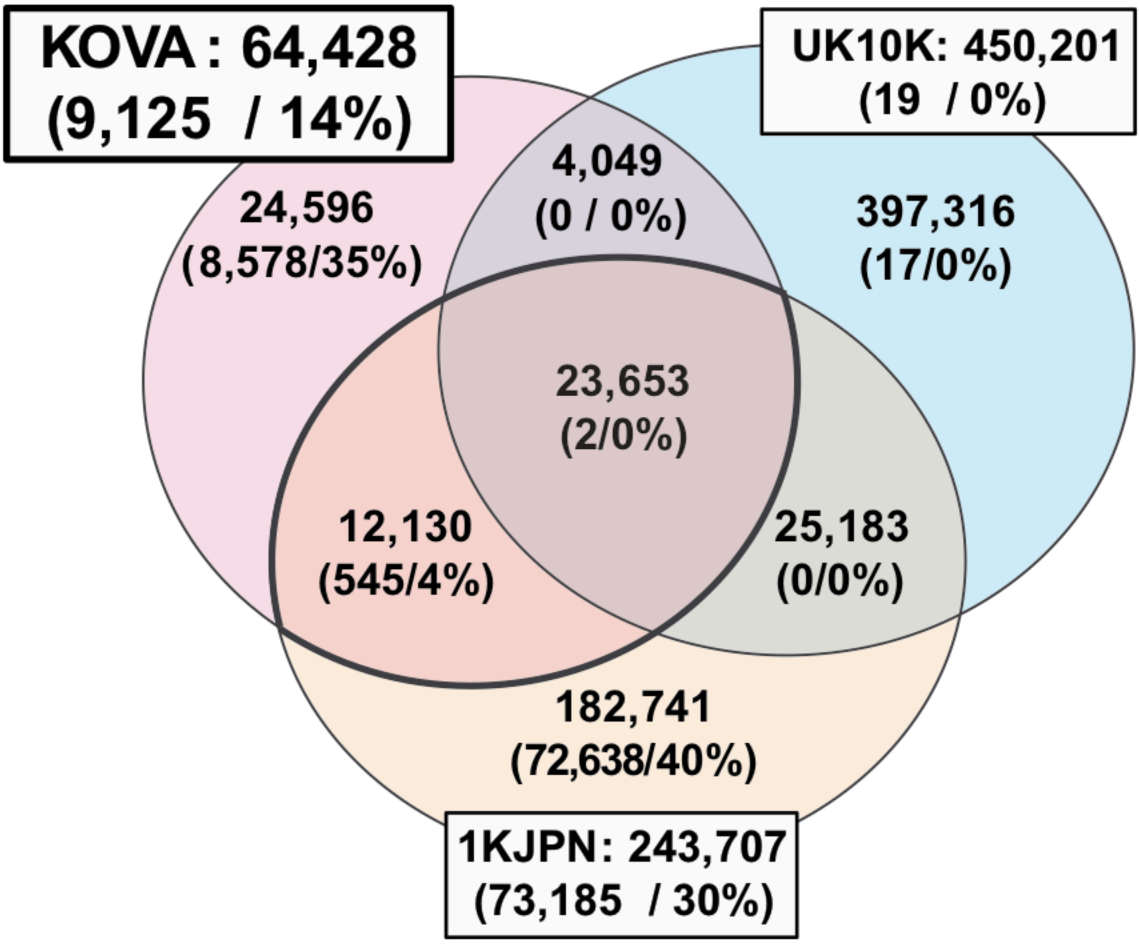

Supplementary Figure S5. Venn diagram of coding variant comparisons among KOVA, Japanese population (1KJPN), and UK10K. Numbers and proportion of novel variants (i.e. not registered in dbSNP build 147) in each area are shown in the parentheses.

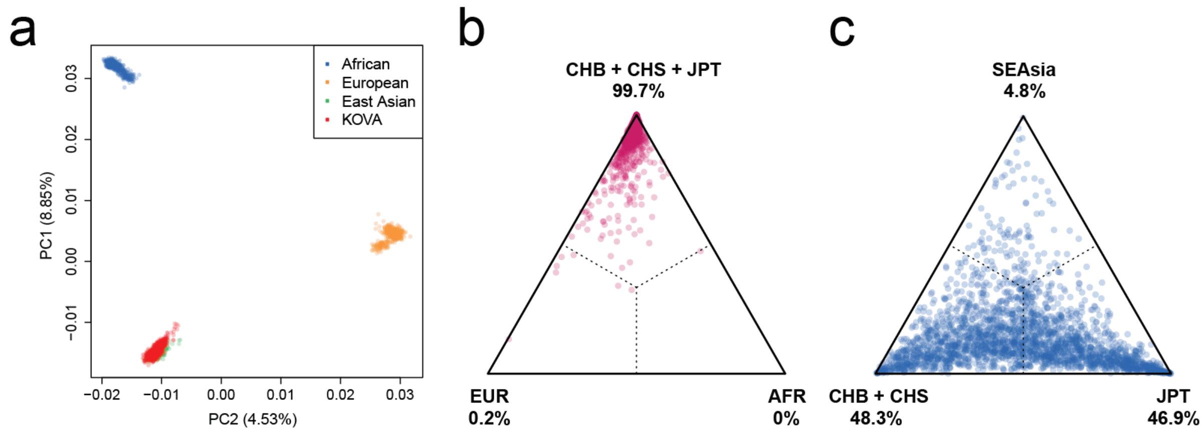

Supplementary Figure S6. Principal component analysis and 0.5Mb-windowed fixation index (F_{ST}) of KOVA and other ethnic groups. (**a**) Principal component analysis of KOVA and other populations of 1000 Genomes Project. 0.5Mb-windowed variant-level F_{ST} between KOVA and (**b**) East Asian, European, and African, and (**c**) Chinese, Japanese, and Southeast Asian.

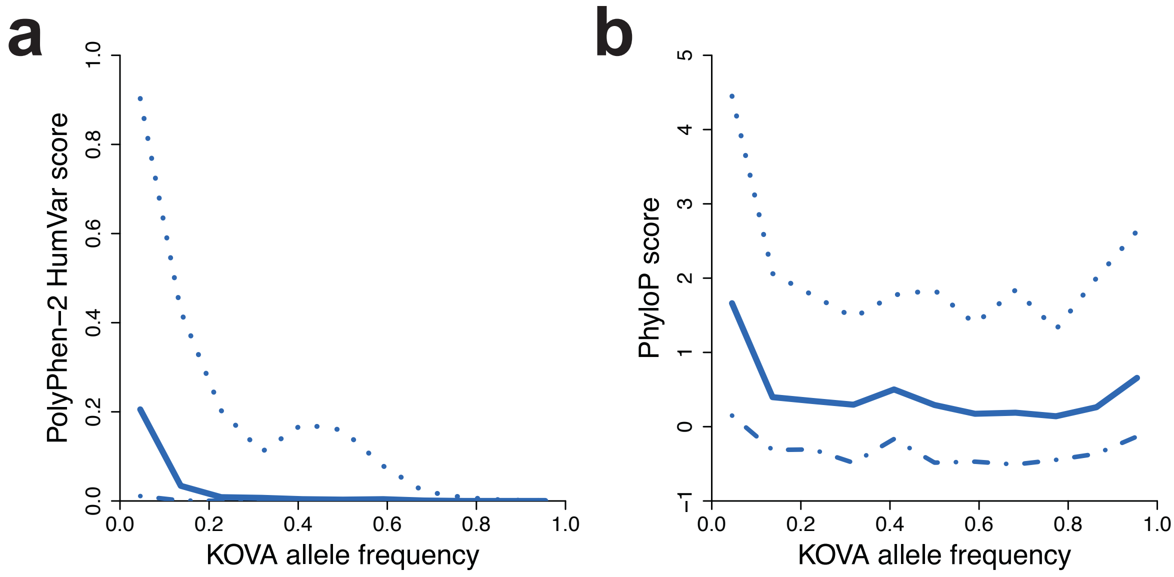

Supplementary Figure S7. Functional analysis of nonsynonymous variants. (**a**) PolyPhen-2 HumVar and (**b**) PhyloP score distributions by allele frequencies.

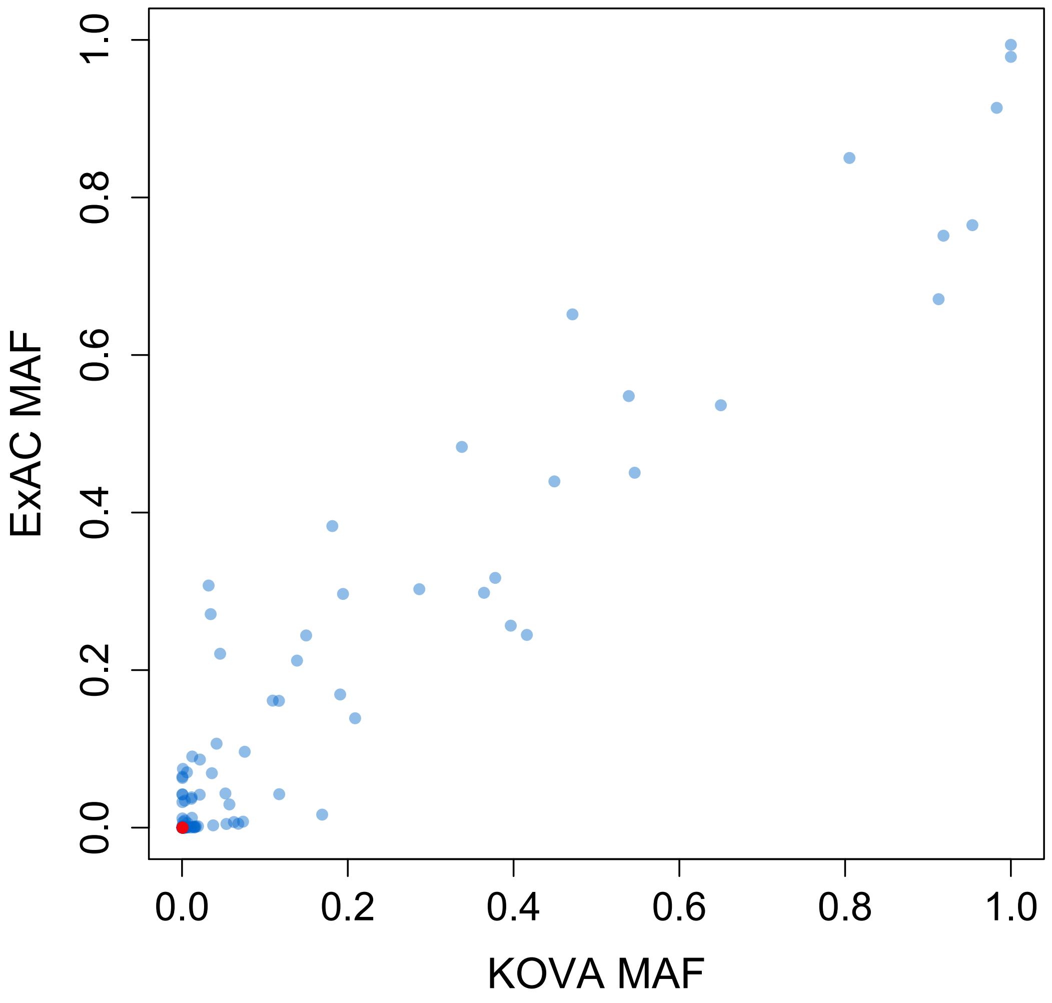

Supplementary Figure S8. Minor allele frequency comparison of variants annotated as pathogenic in ClinVar between KOVA and ExAC. ExAC: Exome Aggregation Consortium, MAF: Minor allele frequency.

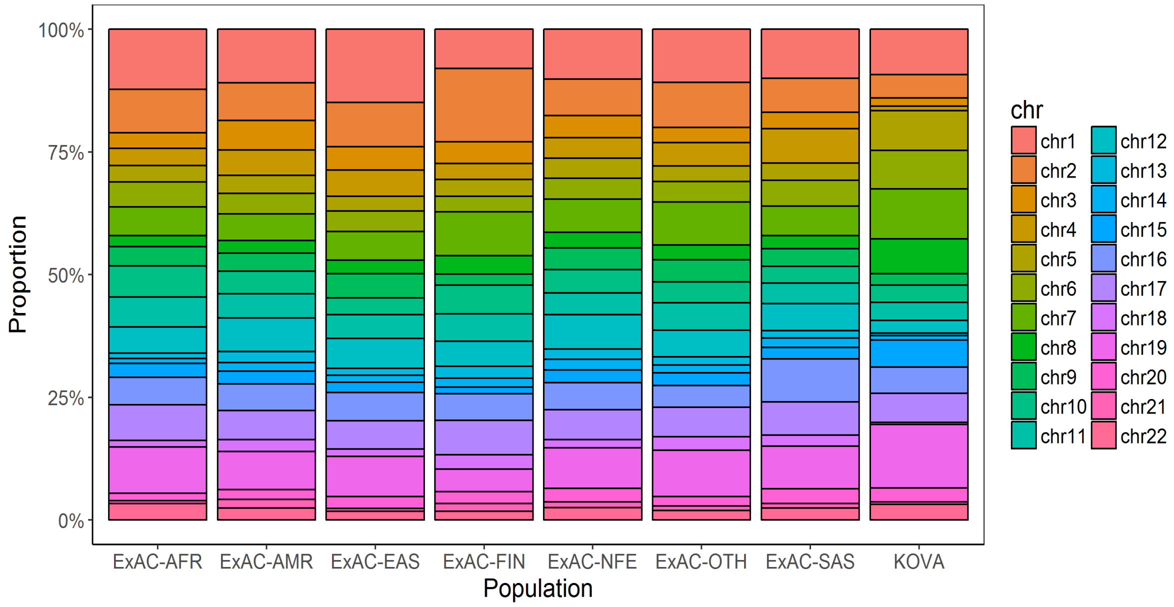

Supplementary Figure S9. CNV count distribution of each chromosome in ExAC and KOVA.

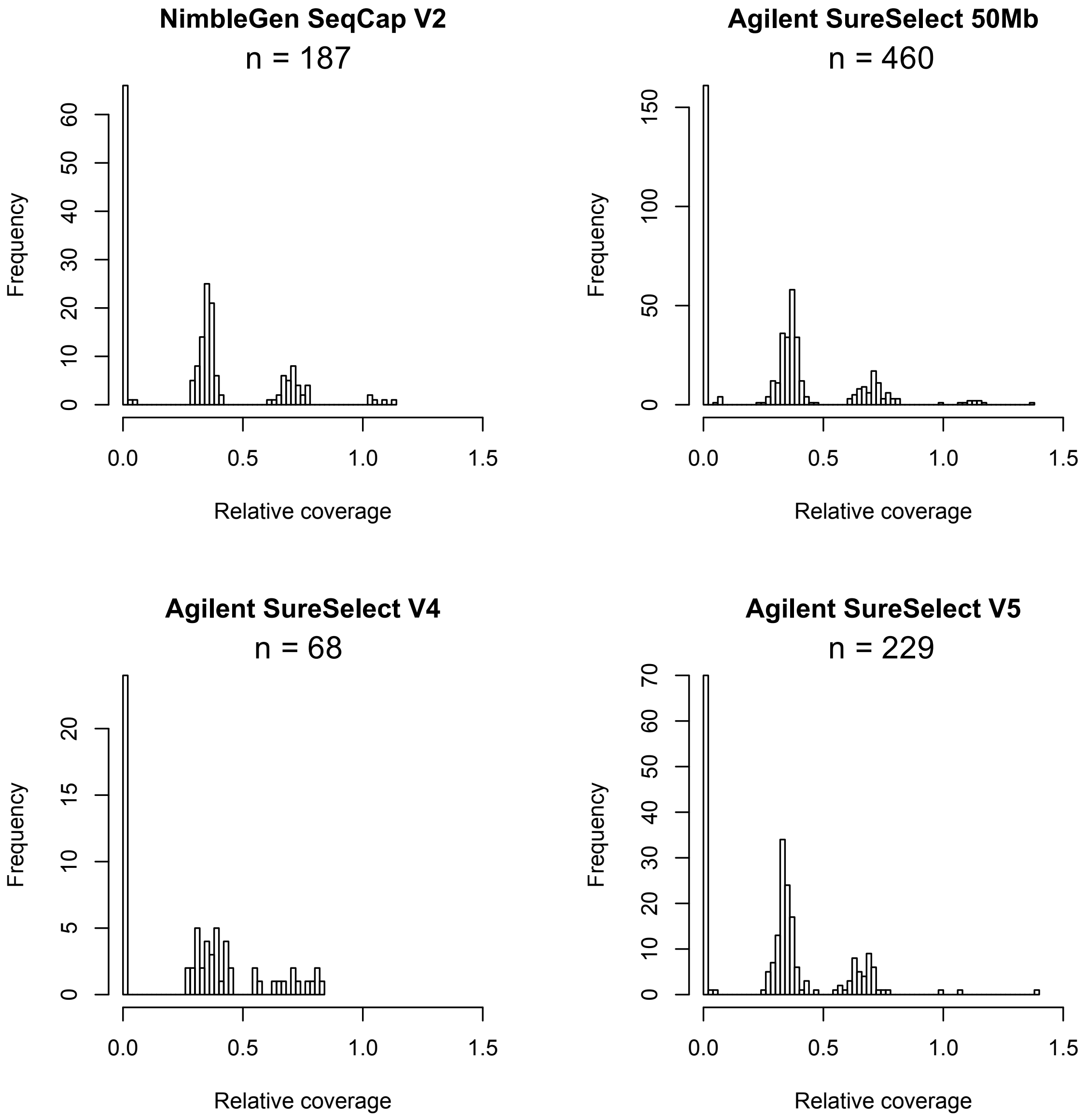

Supplementary Figure S10. Relative coverage of *SIGLEC14* locus to mean coverage of chromosome 19 by exome capture platforms.

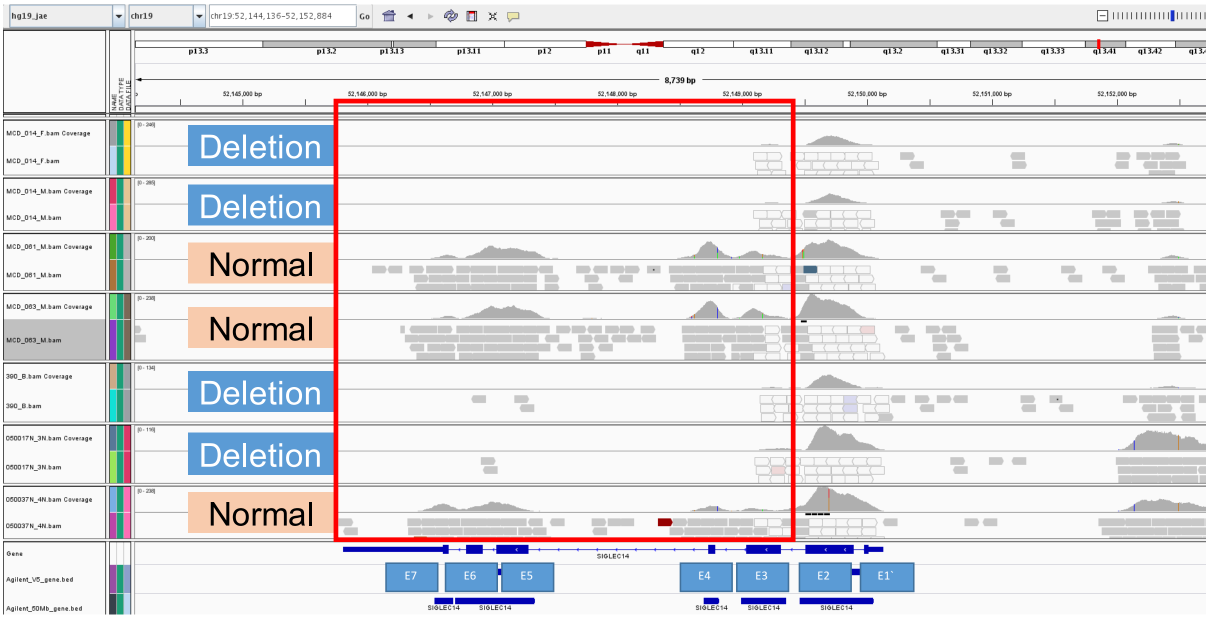

Supplementary Figure S11. Example of IGV snapshot of *SIGLEC14* deletion.

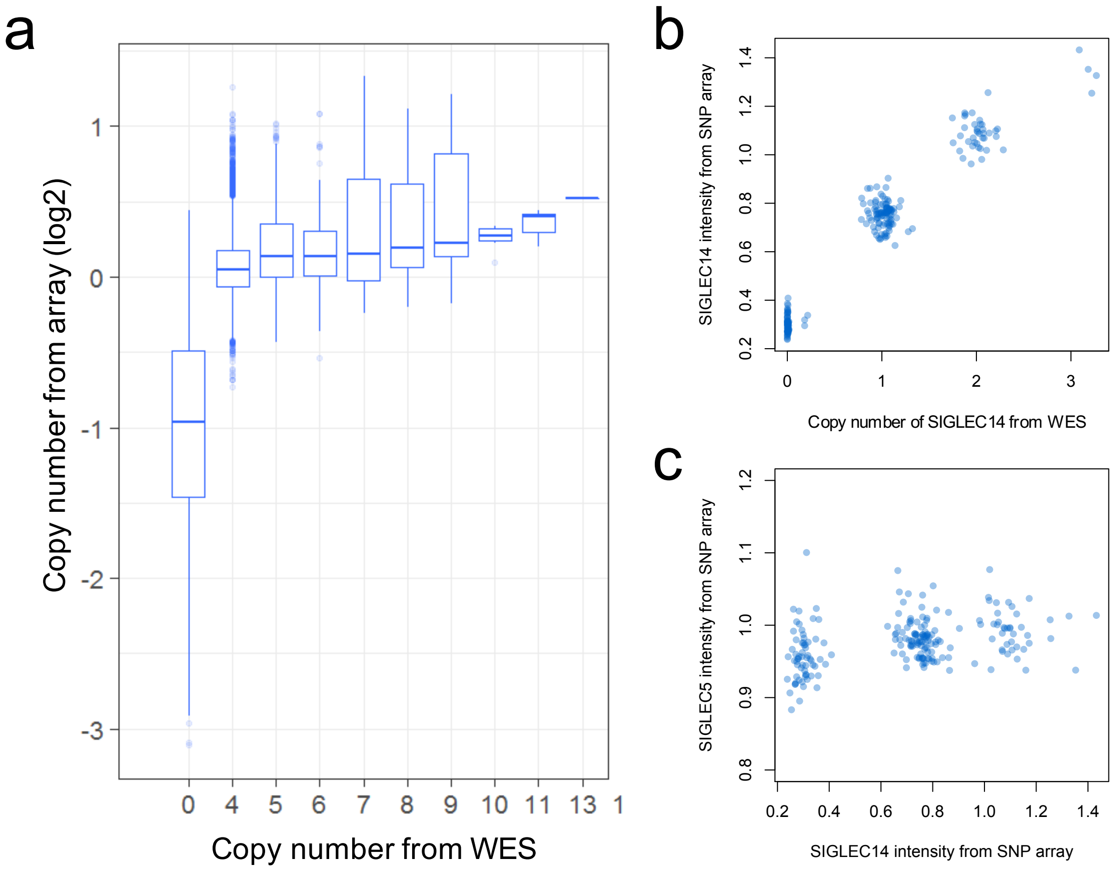

Supplementary Figure S12. KOVA copy number variation validated by SNP array. (**a**) Correlation of copy numbers (CNs) between KOVA and corresponding SNP array (Correlation = 0.43, $P < 2.0 \times 10^{-16}$). (**b**) CNs of *SIGLEC14* estimated by WES data is very well concordant with that by SNP array. (**c**) CNs of *SIGLEC14* and *SIGLEC5* predicted by SNP array.

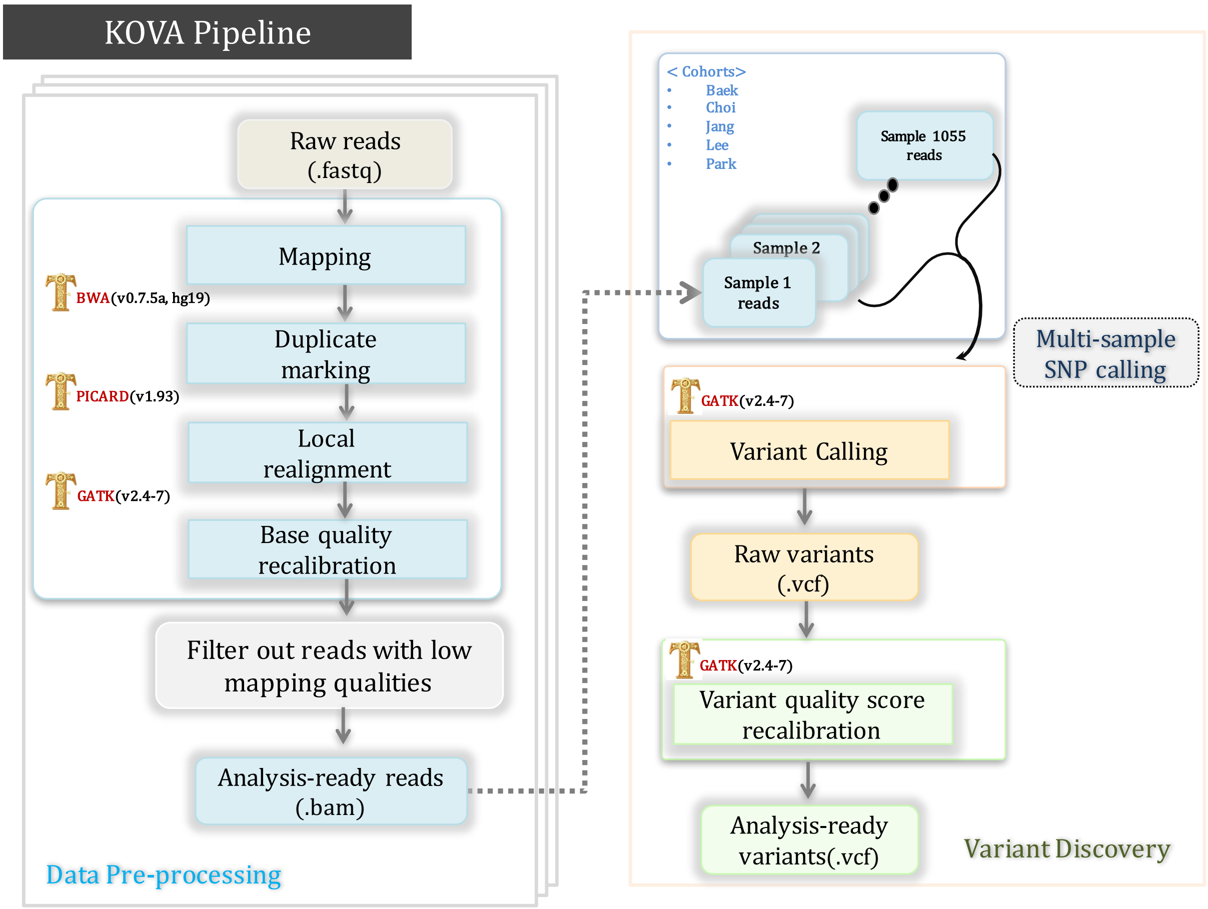

Supplementary Figure S13. Whole exome sequencing pipeline that used in this study.

Supplementary Table S1. Summary of sample cohorts

Group	No. of sample	Sample Information	Capture platform	Mean cov. depth
Baek	237	Lung adenocarcinoma normal #237	Agilent SureSelect 50Mb	54X
Choi	190	Healthy parent of a rare disease pt #166<br>Cancer normal #23<br>Healthy person #1	Roche V2	71X
Jang	123	Healthy person #123	Agilent SureSelect 50Mb	51X
Lee	214	Lung adenocarcinoma normal #127<br>Stomach cancer normal #76<br>Normal #11	Agilent SureSelect 50Mb<br>Agilent SureSelect V4	93X
Park	291	Breast cancer normal #137<br>Healthy parent of a rare disease pt #77<br>Glioblastoma normal #62<br>Colorectal cancer normal #13<br>Healthy family of hearing loss pt#1<br>Healthy person #1	Illumina TruSeq<br>Agilent SureSelect V4<br>Agilent SureSelect V5	109X
Total	1055			75X

Supplementary Table S2. Distribution of Minor Allele Frequency in KOVA

MAF	No. of variants	%	Known	Novel	% novel
Singleton	106,826	36.45	41,491	65,335	61.16
Singleton < MAF ≦ 0.01	85,978	29.34	63,481	22,497	26.17
0.01 < MAF ≦ 0.05	24,108	8.23	23,920	188	0.78
0.05 < MAF ≦ 0.1	11,253	3.84	11,247	6	0.05
0.1 < MAF ≦ 0.2	14,037	4.79	14,033	4	0.03
0.2 < MAF ≦ 0.3	9,803	3.35	9,802	1	0.01
0.3 < MAF ≦ 0.4	7,590	2.59	7,588	2	0.03
0.4 < MAF ≦ 0.5	6,437	2.20	6,437	0	0
0.5 < MAF ≦ 0.6	5,377	1.83	5,375	2	0.04
0.6 < MAF ≦ 0.7	4,797	1.64	4,797	0	0
0.7 < MAF ≦ 0.8	3,965	1.35	3,965	0	0
0.8 < MAF ≦ 0.9	3,861	1.32	3,859	2	0.05
0.9 > MAF	9,017	3.08	9,007	10	0.11
Total	293,049	100.00	205,002	88,047	30.05

MAF; Minor allele frequency

Supplementary Table S3. Summary of copy number variations in each group

Group	# of samples	Average # of amp.	Average # of del.	Amp. mean length (Kb)	Del. mean length (Kb)	Amp. median length (Kb)	Del. median length (Kb)
Baek	211	12	5	9.6	5.9	2.8	2.4
Choi	187	5	3	9.5	4.5	2.9	2.0
Jang	118	11	5	11.2	5.6	2.8	2.8
Lee	199	10	10	10.8	12.6	2.8	3.1
Park	229	12	4	15.1	3.7	1.3	1.0

Supplementary Table S4. Rare variants that were enriched in "tumor-paired normals" compared to "healthy normals" for lung cancer

Chromosome	Position	dbSNP142	Reference	Alternative	Minor allele frequency				
					KOVA:cancer	KOVA:normal	1000GP:EAS	TCGA:LUAD	1000GP:EUR
chr1	7,880,683	rs10462020	T	G	0.014	0.007	0.008	0.162	0.197
chr2	24,439,048	rs3731625	A	G	0.100	0.060	0.001	0.083	0.000
chr3	32,578,505	rs2303857	T	C	0.040	0.026	0.054	0.109	0.098
chr3	45,814,094	rs17279437	G	A	0.012	0.004	0.000	0.066	0.000
chr3	49,736,537	rs77308703	C	T	0.034	0.022	0.000	0.002	0.000
chr3	178,968,634	rs7645550	C	T	0.091	0.060	0.123	0.415	0.371
chr4	108,969,883	rs4365796	G	A	0.026	0.015	0.000	0.002	0.000
chr4	178,256,913	rs7689099	C	G	0.021	0.011	0.026	0.162	0.118
chr5	79,029,594	rs1019762	T	C	0.030	0.004	0.000	0.133	0.002
chr5	141,025,730	rs77115131	T	G	0.043	0.015	0.035	0.024	0.032
chr5	154,346,296	rs3749671	G	A	0.059	0.037	0.053	0.044	0.040
chr5	168,123,398	rs2305993	T	C	0.019	0.004	0.008	0.002	0.001
chr6	26,157,073	rs2298090	A	G	0.026	0.007	0.013	0.007	0.012
chr6	29,395,057	rs16894898	T	C	0.055	0.022	0.030	0.009	0.000
chr6	32,784,676	rs2071554	C	T	0.113	0.071	0.060	0.050	0.050
chr6	52,101,758	rs11465553	C	T	0.014	0.007	0.000	0.044	0.000
chr7	44,104,788	rs61756062	A	C	0.038	0.022	0.039	0.011	0.004
chr7	103,292,112	rs115734214	T	G	0.026	0.015	0.029	0.028	0.032
chr7	138,417,791	rs3807153	A	G	0.045	0.026	0.000	0.039	0.000
chr8	27,362,587	rs57699806	G	A	0.018	0.011	0.061	0.004	0.000
chr8	144,130,643	rs78140118	G	A	0.021	0.011	0.020	0.004	0.000
chr9	136,305,530	rs28647808	C	G	0.022	0.007	0.020	0.092	0.083
chr10	21,134,282	rs41277370	C	G	0.019	0.004	0.001	0.076	0.000
chr10	48,388,228	rs11204213	C	T	0.033	0.015	0.042	0.002	0.001
chr10	94,714,427	rs11187225	C	G	0.012	0.004	0.004	0.107	0.102
chr10	95,441,272	rs11187583	G	A	0.025	0.011	0.034	0.098	0.119
chr11	5,510,688	rs7101919	T	C	0.070	0.041	0.000	0.323	0.007
chr11	102,988,504	rs12146610	A	T	0.012	0.007	0.007	0.061	0.067
chr12	7,842,587	rs2302516	C	G	0.040	0.022	0.059	0.033	0.023
chr12	89,916,811	rs2230283	C	T	0.159	0.105	0.137	0.288	0.353
chr13	97,484,830	rs9516771	A	G	0.012	0.007	0.021	0.068	0.049
chr13	108,863,609	rs1805389	G	A	0.111	0.067	0.100	0.046	0.059
chr14	20,612,763	rs10141025	G	A	0.066	0.041	0.033	0.002	0.000
chr14	22,038,001	rs80295194	C	T	0.056	0.026	0.029	0.002	0.001
chr14	23,549,285	rs3751501	G	A	0.098	0.052	0.139	0.052	0.050
chr14	68,251,991	rs3742885	G	A	0.164	0.108	0.124	0.024	0.018
chr14	94,756,458	rs2232699	A	T	0.034	0.022	0.030	0.002	0.000
chr15	81,234,287	rs16972583	C	G	0.029	0.007	0.015	0.002	0.000
chr15	99,671,765	rs5030698	G	C	0.026	0.011	0.021	0.076	0.059
chr16	56,936,319	rs12708965	C	T	0.030	0.019	0.026	0.022	0.016
chr16	81,077,016	rs2278023	A	G	0.062	0.037	0.049	0.002	0.003
chr16	81,211,496	rs9935113	C	A	0.092	0.060	0.105	0.142	0.171
chr16	89,815,152	rs17233497	G	A	0.029	0.011	0.022	0.090	0.065
chr17	4,352,636	rs34457931	G	A	0.063	0.034	0.069	0.127	0.137
chr17	8,243,598	rs73250854	C	T	0.019	0.011	0.000	0.118	0.000
chr17	47,246,163	rs7224888	T	C	0.065	0.041	0.022	0.087	0.120
chr17	74,019,680	rs78044548	C	T	0.021	0.011	0.037	0.039	0.058
chr18	11,889,467	rs11872520	C	T	0.036	0.015	0.025	0.004	0.003
chr20	3,682,126	rs34924243	C	T	0.015	0.007	0.013	0.063	0.057
chr20	35,507,558	rs3748460	G	A	0.034	0.019	0.037	0.004	0.005
chr21	31,692,277	rs3804007	G	T	0.018	0.007	0.022	0.061	0.053
chr21	46,117,823	rs61745911	G	A	0.037	0.019	0.000	0.028	0.000
chr22	30,888,494	rs17738527	C	T	0.036	0.022	0.040	0.203	0.230
chr22	32,650,200	rs2235171	C	T	0.040	0.015	0.030	0.028	0.037

Supplementary Table S5. Rare variants that were enriched in “tumor-paired normals” compared to “healthy normals” for stomach cancer

Chromosome	Position	dbSNP142	Reference	Alternative	Minor allele frequency				
					KOVA:cancer	KOVA:normal	1000GP:EAS	TCGA:LUAD	1000GP:EUR
chr1	19,566,382	rs3850531	A	G	0.125	0.060	0.056	0.004	0.000
chr1	39,847,730	rs79306726	G	A	0.158	0.097	0.104	0.011	0.003
chr1	75,097,426	rs11210490	G	C	0.171	0.108	0.114	0.438	0.527
chr1	94,564,483	rs6657239	C	T	0.053	0.030	0.015	0.033	0.015
chr1	150,958,836	rs267733	A	G	0.112	0.052	0.026	0.106	0.138
chr1	167,780,071	rs117021474	C	T	0.020	0.007	0.009	0.004	0.001
chr2	70,037,739	rs2228202	C	G	0.118	0.067	0.000	0.007	0.001
chr2	160,310,246	rs10202670	A	G	0.046	0.019	0.013	0.142	0.115
chr3	12,962,074	rs35319679	G	A	0.204	0.127	0.163	0.150	0.136
chr3	52,390,789	rs61734640	C	T	0.032	0.019	0.019	0.033	0.056
chr3	52,429,665	rs419752	C	T	0.033	0.019	0.000	0.036	0.000
chr3	113,285,276	rs2271496	G	A	0.145	0.078	0.090	0.036	0.039
chr3	113,720,517	rs6784095	T	A	0.145	0.093	0.069	0.099	0.089
chr3	190,158,168	rs35161724	G	C	0.013	0.007	0.018	0.125	0.144
chr4	5,830,296	rs34611001	C	T	0.026	0.004	0.022	0.015	0.023
chr4	38,830,116	rs3796508	C	T	0.039	0.022	0.082	0.007	0.002
chr4	96,091,414	rs34585936	C	T	0.046	0.026	0.035	0.011	0.021
chr4	108,969,883	rs4365796	G	A	0.033	0.015	0.000	0.011	0.000
chr5	79,029,594	rs1019762	T	C	0.026	0.004	0.000	0.165	0.002
chr5	94,230,358	rs9885412	C	T	0.086	0.049	0.070	0.140	0.110
chr5	140,558,212	rs2950845	A	C	0.086	0.052	0.091	0.131	0.144
chr5	141,025,730	rs77115131	T	G	0.039	0.011	0.035	0.024	0.032
chr5	154,346,296	rs3749671	G	A	0.072	0.037	0.053	0.040	0.040
chr5	168,123,398	rs2305993	T	C	0.020	0.004	0.008	0.007	0.001
chr6	26,091,179	rs1799945	C	G	0.079	0.030	0.001	0.172	0.000
chr6	26,157,073	rs2298090	A	G	0.026	0.007	0.013	0.018	0.012
chr6	29,395,057	rs16894898	T	C	0.072	0.022	0.030	0.004	0.000
chr6	31,122,502	rs130075	C	T	0.072	0.045	0.038	0.099	0.042
chr6	32,814,942	rs1057149	C	T	0.013	0.007	0.000	0.015	0.000
chr7	44,104,788	rs61756062	A	C	0.066	0.019	0.039	0.011	0.004
chr7	80,427,530	rs1527482	C	T	0.086	0.045	0.063	0.011	0.006
chr7	129,663,496	rs11556924	C	T	0.079	0.026	0.005	0.347	0.000
chr7	138,417,791	rs3807153	A	G	0.053	0.026	0.000	0.040	0.000
chr8	6,794,400	rs28661751	C	G	0.079	0.049	0.067	0.004	0.009
chr8	24,349,417	rs3736281	T	C	0.033	0.015	0.024	0.008	0.013
chr9	2,820,046	rs3736390	G	C	0.013	0.004	0.000	0.004	0.000
chr9	124,065,224	rs2230287	G	A	0.197	0.127	0.122	0.029	0.012
chr9	125,659,716	rs78926480	C	T	0.053	0.015	0.045	0.004	0.003
chr9	136,305,530	rs28647808	C	G	0.020	0.007	0.020	0.062	0.083
chr10	94,714,427	rs11187225	C	G	0.013	0.004	0.004	0.077	0.102
chr10	95,441,272	rs11187583	G	A	0.020	0.011	0.034	0.051	0.119
chr10	105,840,422	rs17116471	T	C	0.105	0.060	0.039	0.004	0.000
chr11	4,945,233	rs35264256	C	T	0.020	0.004	0.008	0.124	0.000
chr11	10,647,995	rs35468145	G	A	0.013	0.007	0.007	0.062	0.038
chr11	32,955,122	rs2297781	A	G	0.105	0.060	0.060	0.044	0.058
chr11	58,034,651	rs7111538	C	T	0.059	0.030	0.040	0.018	0.015
chr11	94,759,494	rs16921260	T	C	0.079	0.045	0.036	0.036	0.039
chr12	6,980,442	rs61733180	G	A	0.045	0.015	0.040	0.004	0.000
chr12	7,842,587	rs2302516	C	G	0.046	0.022	0.059	0.029	0.023
chr12	15,038,703	rs142330429	G	A	0.052	0.015	0.031	0.004	0.004
chr12	57,114,869	rs17118953	T	C	0.013	0.007	0.005	0.026	0.027
chr14	91,671,124	rs2295524	G	A	0.158	0.071	0.212	0.102	0.091
chr15	50,773,787	rs3743044	A	G	0.125	0.082	0.000	0.033	0.000
chr15	99,671,765	rs5030698	G	C	0.045	0.011	0.021	0.091	0.059
chr16	72,184,566	rs35370634	C	T	0.013	0.007	0.022	0.179	0.162
chr16	81,211,496	rs9935113	C	A	0.092	0.060	0.105	0.175	0.171
chr16	84,031,884	rs2292329	G	C	0.112	0.041	0.000	0.022	0.000
chr17	8,243,598	rs73250854	C	T	0.026	0.011	0.000	0.113	0.000
chr17	48,165,148	rs146126922	C	G	0.013	0.007	0.000	0.004	0.000
chr18	11,889,467	rs11872520	C	T	0.026	0.015	0.025	0.007	0.003
chr19	6,187,686	rs17851960	G	A	0.046	0.030	0.029	0.048	0.018
chr19	11,348,960	rs12609039	G	A	0.132	0.056	0.070	0.078	0.052
chr19	15,233,732	rs59102460	G	A	0.138	0.082	0.104	0.007	0.005
chr19	24,310,413	rs12611425	G	C	0.086	0.041	0.000	0.062	0.000
chr20	34,220,755	rs11543244	C	T	0.066	0.030	0.052	0.044	0.052
chr20	60,909,316	rs6062223	C	T	0.046	0.019	0.000	0.026	0.001
chr20	61,907,982	rs11542296	C	T	0.033	0.015	0.011	0.055	0.079
chr21	31,692,277	rs3804007	G	T	0.020	0.007	0.022	0.036	0.053
chr22	23,482,483	rs35211242	G	A	0.046	0.026	0.039	0.175	0.147
chr22	24,761,467	rs204718	G	A	0.039	0.022	0.999	1.000	1.000
chr22	32,650,200	rs2235171	C	T	0.033	0.015	0.030	0.015	0.037
chr22	44,131,786	rs3747203	T	C	0.165	0.101	0.000	0.069	0.000

Supplementary Table S6. ANNOVAR Databases Used for Functional Annotation

Build	Table Name	Table Explanation	Date
hg19	refGene	FASTA sequences for all annotated transcripts in RefSeq Gene	20150322
	snp138	dbSNP with ANNOVAR index files	20140910
	avsnp142	dbSNP142 with allelic splitting and left-normalization	20141228
	avsnp147	dbSNP147 with allelic splitting and left-normalization	20160606
	1000g2014oct (1000g2014oct_all, 1000g2014oct_eur, 1000g2014oct_amr, 1000g2014oct_afr, 1000g2014oct_eas, 1000g2014oct_sas)	alternative allele frequency data in 1000 Genomes Project for autosomes (ALL, AFR (African), AMR (Admixed American), EAS (East Asian), EUR (European), SAS (South Asian)). Based on 201409 collection v5 (based on 201305 alignment) but including chrX and chrY data finally	20141216
	esp6500siv2_all	alternative allele frequency in All subjects in the NHLBI-ESP project with 6500 exomes, including the indel calls and the chrY calls	20141222
	esp6500siv2_aa	alternative allele frequency in African American subjects in the NHLBI-ESP project with 6500 exomes, including the indel calls and the chrY calls	20141222
	esp6500siv2_ea	alternative allele frequency in European American subjects in the NHLBI-ESP project with 6500 exomes, including the indel calls and the chrY calls	20141222
	exac03	ExAC 65000 exome allele frequency data for ALL, AFR (African), AMR (Admixed American), EAS (East Asian), FIN (Finnish), NFE (Non-finnish European), OTH (other), SAS (South Asian)). version 0.3. Left normalization done	20150729
	popfreq_all_20150413	A database containing all allele frequency from 1000G, ESP6500, ExAC and CG46	20150413
	cosmic70	COSMIC database version 68 on WGS data	20140911
	clinvar_20150629	CLINVAR database with Variant Clinical Significance (unknown, untested, non-pathogenic, probable-non-pathogenic, probable-pathogenic, pathogenic, drug-response, histocompatibility, other) and Variant disease name	20150724
	nci60	NCI-60 human tumor cell line panel exome sequencing allele frequency data	20130724
	ljb26_all	whole-exome SIFT, PolyPhen2 HDIV, PolyPhen2 HVAR, LRT, MutationTaster, MutationAssessor, FATHMM, MetaSVM, MetaLR, VEST, CADD, GERP++, PhyloP and SiPhy scores from dbNSFP version 2.6	20140925
